# Molecular heterogeneity of BCL2/MYC double expressor lymphoma underlies sensitivity to histone deacetylase inhibitor

**DOI:** 10.1002/ctm2.1691

**Published:** 2024-05-29

**Authors:** Zi‐Yang Shi, Ying Fang, Peng‐Peng Xu, Hong‐Mei Yi, Jian‐Feng Li, Yan Dong, Yue Zhu, Meng‐Ke Liu, Di Fu, Shuo Wang, Qing Shi, Rong Shen, Hui‐Juan Zhong, Chao‐Fu Wang, Shu Cheng, Li Wang, Feng Liu, Wei‐Li Zhao

**Affiliations:** ^1^ Shanghai Institute of Hematology, State Key Laboratory of Medical Genomics, National Research Center for Translational Medicine at Shanghai, Shanghai Ruijin Hospital Shanghai Jiao Tong University School of Medicine Shanghai China; ^2^ Department of Pathology, Shanghai Ruijin Hospital Shanghai Jiao Tong University School of Medicine Shanghai China; ^3^ Pôle de Recherches Sino‐Français en Science du Vivant et Génomique Laboratory of Molecular Pathology Shanghai China

Dear Editor,

Double expressor lymphoma (DEL) overexpressing BCL2 and MYC protein is an unfavourable subgroup of diffuse large B‐cell lymphoma (DLBCL).^1^ Aiming to investigate heterogeneity and therapeutic implications, we discovered subtypes of DEL with unique characteristics, and deciphered the mechanism underlying histone deacetylase inhibition on DEL.

In this cohort, 157 patients were DEL, and 417 were non‐DEL (160 were double negative for BCL2 and MYC as DNL). The 5‐year progression‐free survival rate of DEL was 37.1%, and overall survival rate was 55.7% (median follow‐up as 45.7 months). Unsupervised clustering analysis^2^ of RNA sequencing (RNA‐seq) data of 157 DEL patients revealed three subtypes (Figures 1A, S1A–C; Table S1): 48 patients of cluster 1 (C1), 63 of cluster 2 (C2) and 46 of cluster 3 (C3). C1 showed similar clinical features as DNL, while C2 and C3 showed high‐risk features (Figure S2; Tables S2 and S3). Among 93 DEL and 117 DNL receiving standard rituximab‐based immunochemotherapy, when compared to DNL, C1, C2 and C3 showed an inferior survival (Figure 1B). As classified by cell‐of‐origin‐^3^ and genetic‐based^4^ classifications, activated B‐cell (ABC)–DLBCL was predominant in C2 and C3, while C1 comprised with ABC, germinal centre B‐cell and unclassified (Figure 1C; Table S3). Meanwhile, each DEL subtype included multiple genetic subtypes, forming a diversified feature (Figure 1D). *PIM1* and *MYD88* mutations were significantly higher in three DEL subtypes than in DNL. C1 was characterised by *KMT2D* mutations. C2 and C3 shared increased *CD79B* mutations (Figure 1E).

**FIGURE 1:**
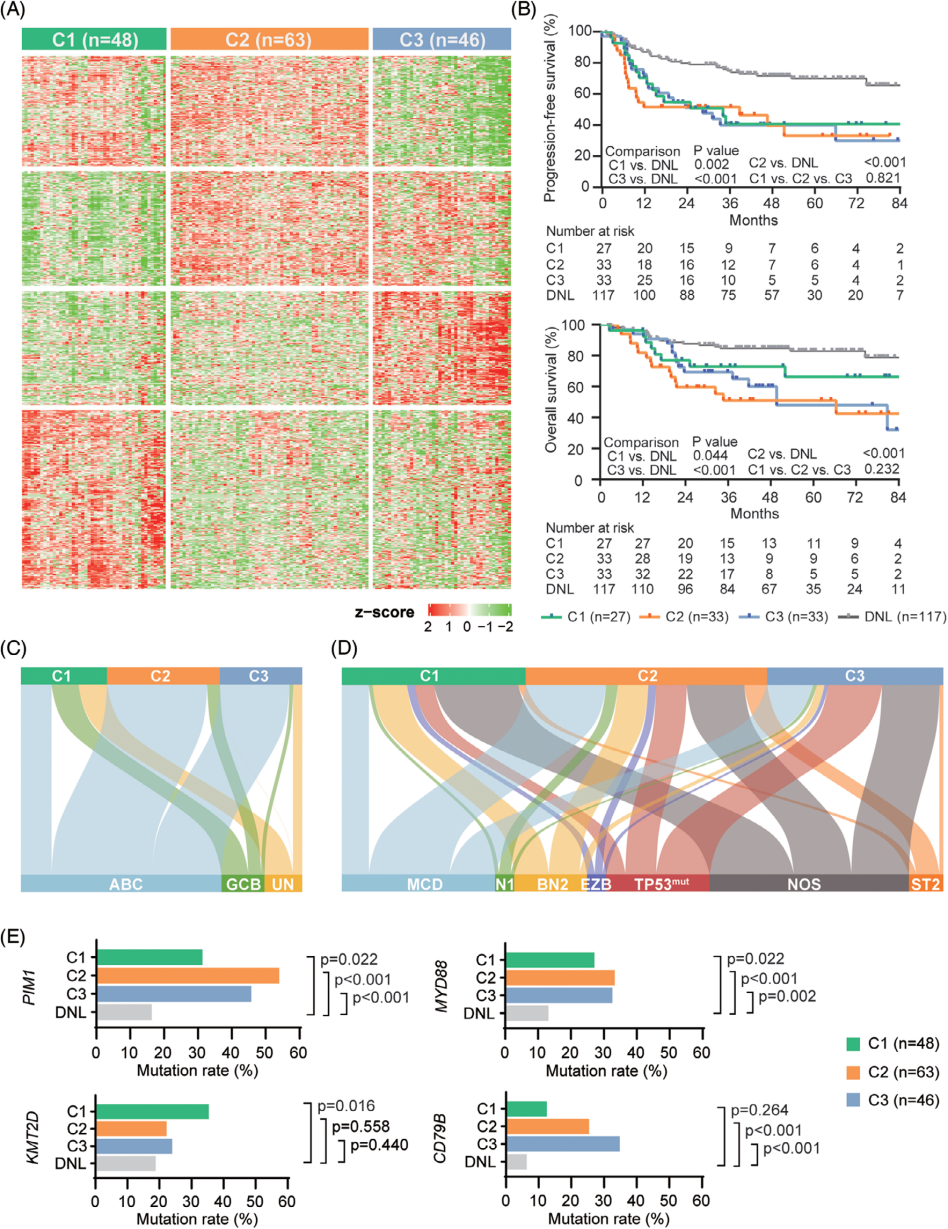
**Identification of DEL subtypes and their clinicopathological features**. (A) Heatmap of unsupervised clustering of gene expression profiles from 157 DEL patients. Heatmap expression level is in *z*‐score. (B) Kaplan–Meier survival curves of PFS (upper panel) and OS (lower panel) in patients treated with standard R‐CHOP, stratified by three DEL subtypes and DNL. *p* values comparing each subtype with DNL and comparing among the three DEL subtypes are shown. The numbers at risk on the time axis are given. (C) Relationship between COO‐based classifications and DEL subtypes. (D) Relationship between genetic‐based classifications and DEL subtypes. (E) Comparison of different prevalence of frequently mutated genes between each DEL subtype and DNL. *p* values calculated with chi‐square tests were shown. AA, Ann Arbor; ABC, activated B‐cell; COO, cell‐of‐origin; DEL, double expressor lymphoma; DNL, double negative lymphoma; GCB, germinal centre B‐cell; IPI, international prognostic index; LDH, lactic dehydrogenase; NOS, not otherwise specified; OS, overall survival; PFS, progression‐free survival; R‐CHOP, rituximab, cyclophosphamide, doxorubicin, vincristine and prednisone; *TP53* ^mut^, *TP53* mutant; UN, unclassified.

When conducting gene set enrichment analysis (GSEA), all three subtypes showed upregulation of NF kappa‐B (NF‐κB) pathway, as compared to DNL (Figure 2A), but differed in upstream biological functions (Figure 2B). Core enrichment genes between C1 and DNL were annotated to T‐cell receptor (TCR) pathway, while both C2 and C3 showed upregulation of B‐cell receptor (BCR) pathway. Pivotal genes in TCR signalling of C1 and BCR signalling of C2 both had a close connection with NF‐κB; while pivotal genes in BCR signalling of C3 had less connection with NF‐κB, suggesting a non‐canonical pattern for BCR signalling in C3 (Figure 2C).

**FIGURE 2:**
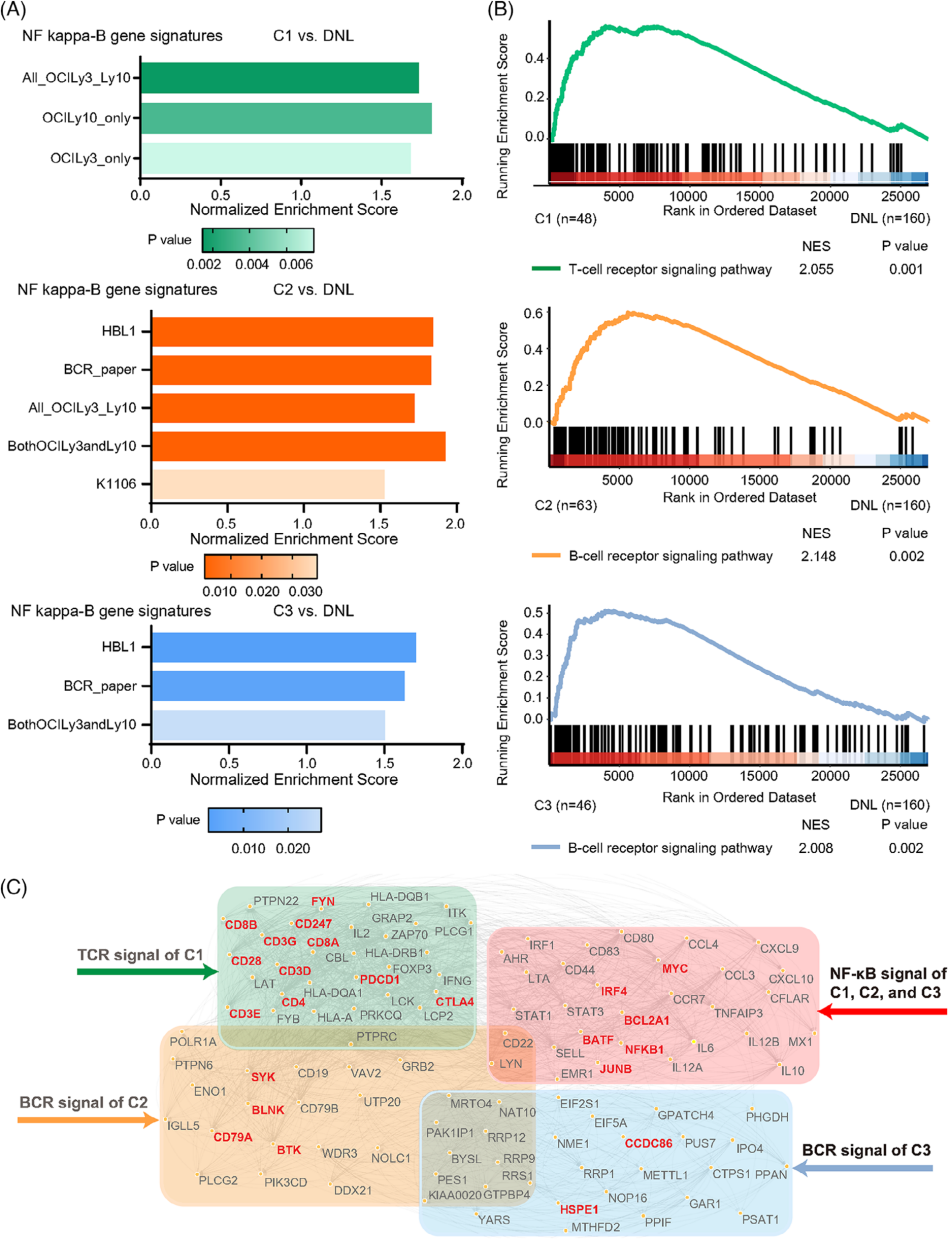
**Profile of oncogenic signalling pathways according to DEL subtypes**. (A) Gene set enrichment analysis identified NF‐κB signalling pathway as upregulated in C1 (upper panel), C2 (middle panel) and C3 (lower panel). (B) Gene set enrichment analysis identified TCR signalling pathway as upregulated in C1 (upper panel), and BCR signalling pathway as upregulated in C2 (middle panel) and C3 (lower panel). (C) Protein–protein interaction showed the core genes and the relationship of the key pathways in each DEL subtype. Footnote: “All_OCILy3_Ly10” and other similar labels on the left *Y*‐axis of the three panels of A represent the names of different NF‐kappa B gene sets obtained from a published online database (https://lymphochip.nih.gov/signaturedb/). BCR, B‐cell receptor; DEL, double expressor lymphoma; DNL, double negative lymphoma; NES, normalised enrichment score; NF‐κB, NF‐kappa B; TCR, T‐cell receptor.

Tumour microenvironment (TME) showed that C1 exhibited a uniquely higher infiltration of CD8^+^T‐cells and M2 macrophages (Figure 3A). Compared between CD8^+^‐high and CD8^+^‐low groups (by median), *PD‐1*, *LAG‐3* and *TIM‐3* were substantially more expressed in CD8^+^‐high group, indicating an exhausted pattern (Figure 3B). In vitro co‐culture system, DLBCL cell line SU‐DHL‐4 with overexpression of BCL2 and MYC were transfected with plasmid containing KMT2D (wild type or *KMT2D* ^R5432Q^), representing typical C1. Flow cytometry showed that, compared to *KMT2D* ^wt^ cells, total CD8^+^T, LAG‐3^+^CD8^+^T, PD‐1^+^CD8^+^T‐cells and M2‐subtype macrophages were considerably higher in *KMT2D* ^mut^ cells (Figures 3C and S3A). Histone deacetylase inhibitor tucidinostat plus doxorubicin led to a significant drop in LAG‐3 and CD8 double positive T‐cells, and M2‐subtype macrophages in *KMT2D* ^mut^ cells, while such changes were not observed in *KMT2D* ^wt^ cells (Figure 3D). Assay for transposase‐accessible chromatin using sequencing (ATAC‐seq) displayed less chromatin accessibility in *KMT2D* ^mut^ cells after dual treatment, while the profile of *KMT2D* ^wt^ cells remained similar (Figure 3E). Downregulated motifs in *KMT2D* ^mut^ cells after treatment were enriched in transcriptional factors (TFs) mediating TCR and NF‐κB pathways (Figure 3F), and in TFs of NR4A family (Figure 3G), the latter of which acted as a bridge between TCR activation and CD8^+^T‐cell exhaustion.^5^ Among them, NR4A2 was also highly expressed in C1 patients (Figure S3B). Those results indicated dysregulated TME as the core feature of C1: *KMT2D* mutations activated TCR signalling, and overexpression of NR4A family TFs, in turn exacerbated CD8^+^T‐cell exhaustion and M2‐subtype macrophages infiltration; and tucidinostat plus doxorubicin could target immune cell dysfunction of TME in C1.

**FIGURE 3:**
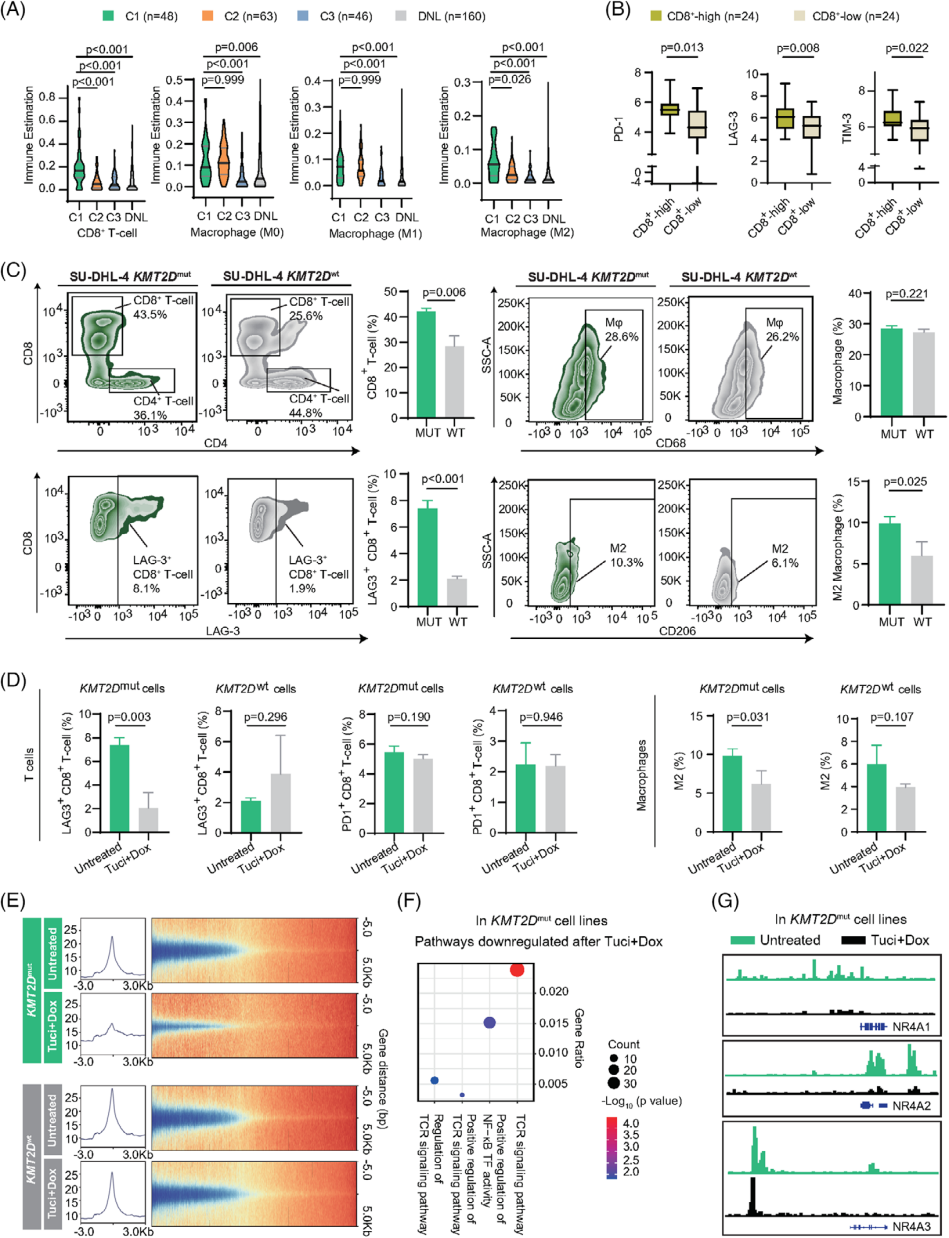
**Tumour microenvironment alterations in C1 subtype of DEL**. (A) Infiltration level of CD8^+^T‐cells and macrophages identified in C1, C2, C3 and DNL patients. Differences calculated by Dunn's multiple comparison test. (B) Boxplots showing the differential expression of inhibitory immune checkpoint markers across CD8^+^‐high or CD8^+^‐low groups. (C) Flow cytometry analysis of proportion of CD8^+^T‐cells, LAG‐3^+^CD8^+^T‐cells, macrophages and M2‐subtype macrophages. Co‐culture of PBMC and SU‐DHL‐4 *KMT2D* ^mut^ cells, and co‐culture of PBMC and SU‐DHL‐4 *KMT2D* ^wt^ cells. (D) Bar plots of proportions of LAG‐3^+^CD8^+^T‐cells, PD‐1^+^CD8^+^T‐cells and M2‐subtype macrophages in PBMC co‐cultured with SU‐DHL‐4 *KMT2D* ^mut^ cell lines or SU‐DHL‐4 *KMT2D* ^wt^ cell lines with tucidinostat (5 µM) and doxorubicin (200 nM) treatment for 48 h. Assays were set up in triplicate. Summarised as the mean ± SD (*n* = 3). (E) Density heatmap of RPKM‐normalised ATAC‐seq signals via semi‐supervised *k*‐means analysis in SU‐DHL‐4 *KMT2D* ^mut^ and SU‐DHL‐4 *KMT2D* ^wt^ cells, before and after tucidinostat and doxorubicin treatment. (F) Downregulated pathways in SU‐DHL‐4 *KMT2D* ^mut^ cells detected by ATAC‐seq after tucidinostat and doxorubicin treatment. (G) Peaks of NR4A family TFs (NR4A1, NR4A2 and NR4A3) were downregulated after tucidinostat and doxorubicin treatment in SU‐DHL‐4 *KMT2D* ^mut^ cells. ATAC‐seq, assay for transposase accessible chromatin sequencing; COO, cell‐of‐origin; DEL, double expressor lymphoma; DNL, double negative lymphoma; MUT, mutant; Mφ, macrophage; PBMC, peripheral blood mononuclear cell; RPKM, reads per kilobase per million mapped reads; SD, standard deviation; TFs, transcriptional factors; Tuci + Dox, tucidinostat and doxorubicin; WT, wild type.

Fresh patient biopsy samples were used to maintain patient‐derived xenograft (PDX) models (passage 3–5),^6^ and tumour were formed in C2, C3 and non‐DEL (Figure 4A); no sample successfully gave rise to PDX models in C1, in consistent with the results that TME was indispensable in C1. The C2‐, C3‐ and DNL‐PDX models were subsequently treated with tucidinostat and doxorubicin. ATAC‐seq and RNA‐seq were performed on biopsy specimen before or 14 days after treatment. GSEA exhibited corresponding results in patients (Figure 2A,B) and PDX models (Figure S4A,B), with NF‐κB, and BCR pathways activated in C2 and C3. ATAC‐seq revealed three differentially accessible regions in C2 and C3, involving in positive regulation of I‐kappa B kinase/NF‐κB and BCR pathways (Figure 4B). The significantly variable motifs differ DEL‐PDXs from DNL‐PDXs could be divided into several groups (Figure 4C). Motifs of AP‐1 family were upregulated in C2 and C3, in line with our previous findings.^6^ Motifs of REL family were enriched in C2, functioning as subunits of NF‐κB complex in haematological malignancies.^7^ And motifs of POU family, involving in NF‐κB activation,^8^ were enriched in C3. Among them, REL (TF of REL family TF) and POU5F1B (TF of POU family) expression levels were high in C2 and C3 patients, respectively (Figure 4D). Target genes of REL and of POU5F1B were verified to be involved in BCR pathways, and upregulated in C2 and C3 patients, respectively (Figure 4D). BCR‐related and REL‐targeted genes of C2 played a critical role in malignant B‐cell activation, bridging BCR‐associated kinase with downstream pathways.^7^ BCR‐related and POU5F1B‐targeted genes of C3 were related to MAPK cascade and cell cycle progression.^9^ After treatment, BCR and NF‐κB pathways were downregulated in both subtypes (Figure S4C); and normalised ATAC‐seq tracks of REL and POU5F1B decreased in C2 and C3, respectively (Figure 4E). Quantitative real‐time PCR detected downtrends of certain target genes after treatment in C2‐ and C3‐PDXs (Figure 4F). Those results revealed the features of C2 and C3, and demonstrated the effect of tucidinostat plus doxorubicin on different oncogenic TFs and signalling networks in the two subtypes. Overall, distinct molecular heterogeneity and complex TME in DEL were major contributors to resistance to standard R‐CHOP, but a broad‐spectrum sensitivity to tucidinostat plus doxorubicin in vitro or in vivo.

**FIGURE 4:**
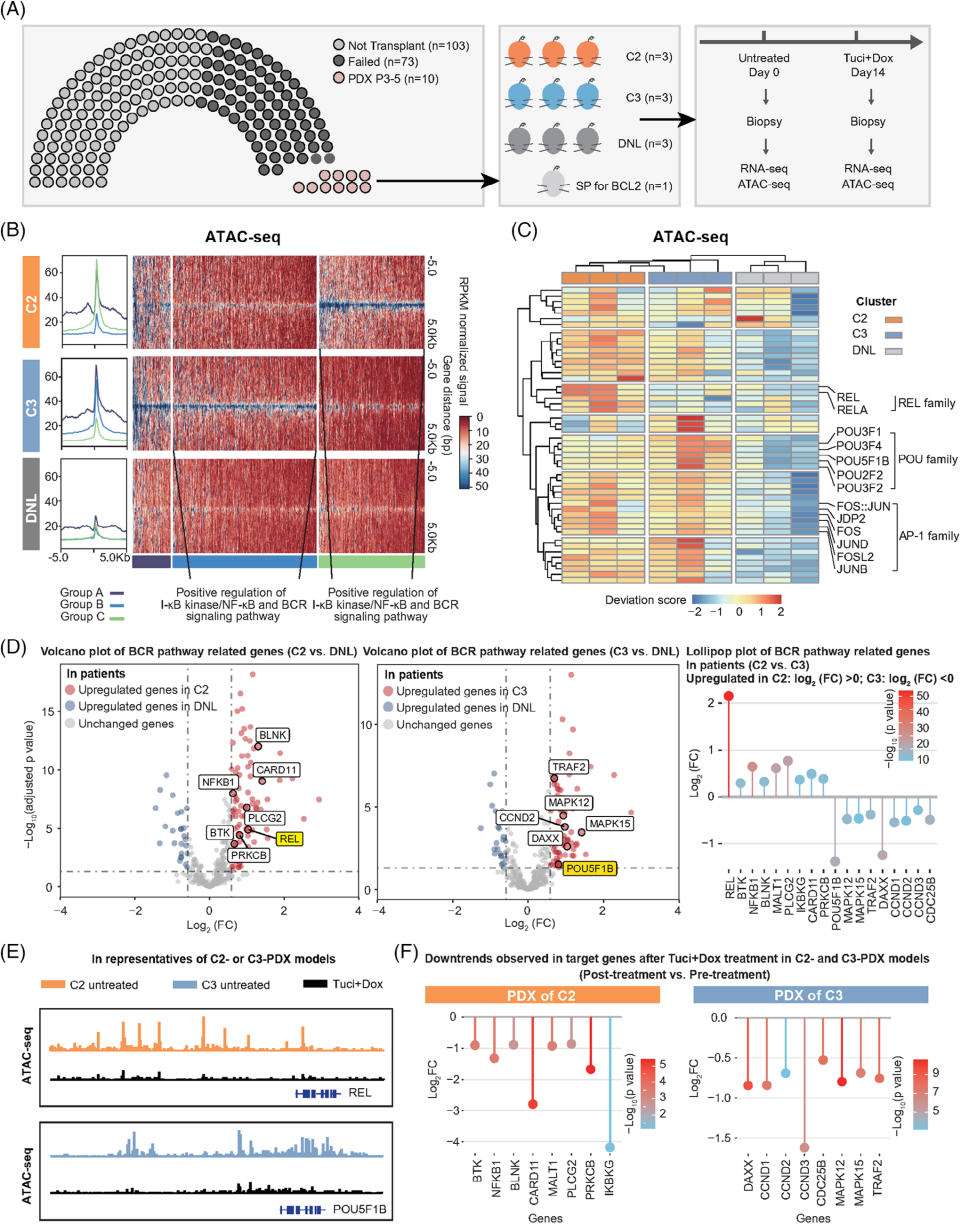
**Oncogenic transcriptional factors and signalling networks in C2 and C3 subtypes of DEL**. (A) Overview of the experiment workflow in C2‐, C3‐ and DNL‐PDX models. (B) Density heatmap of RPKM‐normalised ATAC‐seq signals via semi‐supervised *k*‐means analysis. GO terms associated with these peaks are shown. (C) Hierarchical clustering of ChromVAR motif deviation scores. (D) Volcano plots of gene expression patterns involved in BCR signalling pathway (left panel: C2 vs. DNL; middle panel: C3 vs. DNL). Scattered points represent individual genes. The *X*‐axis is the log_2_FC, and the *Y*‐axis is the −log_10_ (adjusted *p* value). Red dots are genes significantly overexpressed in C2 or C3, and blue dots are genes significantly overexpressed in DNL. Lollipop plot of BCR pathway‐related genes (right panel: C2 vs. C3). Genes relatively upregulated in C2 (log_2_ [FC] > 0), and relatively upregulated in C3 (log_2_ [FC] < 0) were shown. (E) View of ATAC‐seq tracks in pre‐ and post‐treatment PDX models of C2 and C3 at the loci of indicated genes (REL in C2 and POU5F1B in C3). (F) Genes that were downregulated after tucidinostat and doxorubicin treatment in C2‐ and C3‐PDX models, detected by quantitative RT‐PCR. ATAC‐seq, assay for transposase accessible chromatin sequencing; BCR, B‐cell receptor; COO, cell‐of‐origin; DEL, double expressor lymphoma; DNL, double negative lymphoma; FC, fold change; GO, gene ontology; PDX, patient‐derived xenograft; quantitative RT‐PCR, quantitative real‐time PCR; RPKM, reads per kilobase per million mapped reads; SP, single positivity.

In conclusion, this is the first study using multi‐omics data to identify robust DEL subtypes, and to interpret the therapeutic mechanism of epigenetic agents,^10^ providing ideas for optimising the current treatment paradigms in DEL.

## AUTHOR CONTRIBUTIONS

Zi‐Yang Shi, Ying Fang, Peng‐Peng Xu and Hong‐Mei Yi collected and analysed the data, performed the experiments, interpreted the results and wrote the manuscript. Yan Dong, Shuo Wang, Yue Zhu and Meng‐Ke Liu prepared biological samples, performed the experiments and provided executive support. Jian‐Feng Li and Di Fu carried out the sequencing, gave technical support and provided data surveillance. Shu Cheng, Rong Shen, Qing Shi and Hui‐Juan Zhong recruited patients and gathered detailed clinical information for the study. Hong‐Mei Yi and Chao‐Fu Wang reviewed the pathological data. Feng Liu, Li Wang and Wei‐Li Zhao designed and supervised the research, conceived the study and wrote the manuscript. All authors read and approved the final manuscript.

## CONFLICTS OF INTEREST STATEMENT

The authors declare no conflicts of interest.

## ETHICS STATEMENT

The study was approved by the Institutional Review Board of Ruijin Hospital, and informed consent was obtained in accordance with the Declaration of Helsinki. Animals were used according to the ARRIVE guidelines and the protocols approved by Ruijin Hospital Animal Care and Use Committee.

## CONSENT INFORMATION

All the authors have agreed to publish this manuscript.

## Supporting information

Supporting Information

## Data Availability

The data presented in this study are available on reasonable request from the corresponding author.
