# Supplementary material for: Molecular heterogeneity of BCL2/MYC double expressor lymphoma underlies sensitivity to histone deacetylase inhibitor
Source: Clin Transl Med. 2024 May 29;14(6):e1691. doi: 10.1002/ctm2.1691 (PMC11136694; doi:10.1002/ctm2.1691)
Supplement: Supplementary file 1 — Supporting Information [file CTM2-14-e1691-s001.docx]

**Supporting Information for**

**Molecular heterogeneity of BCL2/MYC double expressor lymphoma underlies sensitivity to histone deacetylase inhibitor**

Zi-Yang Shi,^†^ Ying Fang,^†^ Peng-Peng Xu,^†^ Hong-Mei Yi,^†^ Jian-Feng Li, Yan Dong, Yue Zhu, Meng-Ke Liu, Di Fu, Shuo Wang, Qing Shi, Rong Shen, Hui-Juan Zhong, Chao-Fu Wang, Shu Cheng, Li Wang,^*^ Feng Liu,^*^ Wei-Li Zhao^*^

†These authors contributed equally to this study.

*Correspondence to: Wei-Li Zhao, e-mail: zhao.weili@yahoo.com; Feng Liu, e-mail: lf12034@rjh.com.cn; Li Wang, e-mail: wl_wangdong@126.com; Shanghai Institute of Hematology; State Key Laboratory of Medical Genomics; National Research Center for Translational Medicine at Shanghai; Shanghai Ruijin Hospital, Shanghai Jiao Tong University School of Medicine, 197 Rui Jin Er Road, 200025 Shanghai, China, Tel: 0086-21-64370045; Fax: 0086-21-64743206.

This file includes:

Supplementary Methods

Supplementary Figures: S1 to S4

Supplementary Tables: S1 to S5

**Supplementary Methods**

**Patient cohorts and study approval**

A total of 590 patients with newly diagnosed diffuse large B-cell lymphoma (DLBCL) in recent two years were included in this study. Histological diagnoses were reviewed by two pathologists (HMY and CFW). Among all 590 patients, 16 (2.7%) were double hit lymphoma (DHL) with concurrent *MYC* and *BCL2* or *BCL6* rearrangements, 157 (26.6%) were double expressor lymphoma (DEL), and 417 (70.7%) were non-DEL. Moreover, among non-DEL patients, 195 (46.8%) showed single positivity (SP) for BCL2, 62 (14.9%) showed SP for MYC, and 160 (38.4%) were double negative for BCL2 and MYC (designated as double negative lymphoma [DNL]). In this study, we focused on molecularly subtyping the 157 DEL and used the 160 DNL as the outlier group for comparison. Among them, 93 of 157 DEL and 117 of 160 DNL received standard R-CHOP (rituximab, cyclophosphamide, doxorubicin hydrochloride, vincristine sulfate, and prednisone) immunochemotherapy.

The study was approved by the Institutional Review Board of Ruijin Hospital, and informed consent was obtained in accordance with the Declaration of Helsinki.

**Immunohistochemistry (IHC) and** **fluorescence in situ hybridization (FISH)**

Immunohistochemistry (IHC) was performed on 5 μm paraffin sections using antibodies against CD10, BCL6, MUM1, CD5, BCL2 (DAKO, Glostrup, Denmark), and MYC (ZSGB-BIO, Beijing, China). The cell-of-origin (COO) phenotypes were determined by the Hans algorithm.^1^ In tumor cells, the cut-off value of BCL2 positivity in the cytoplasm was 50%, and that of nuclear MYC positivity was 40%.^2,3^ Fluorescence in situ hybridization (FISH) was performed to identify chromosomal breakpoints affecting *MYC*, *BCL2* or *BCL6* loci. Lymphomas positive for both BCL2 and MYC proteins (assessed by IHC) and without BCL2 and MYC rearrangements (assessed by FISH) were defined as DEL in our study, according to the 2016 revision of lymphoid neoplasm classification.^2^

**DNA sequencing and** **molecular classification**

Genomic DNA was extracted from formalin-fixed paraffin-embedded (FFPE) tumor tissue of all 590 patients using a QIAamp DNA Mini Kit or GeneRead DNA FFPE Tissue Kit (Qiagen, Hilden, Germany) respectively. By subjecting qualified samples to DNA sequencing, whole genome sequencing (WGS) was performed on 167 patients, whole exome sequencing (WES) on 118 patients, and targeted sequencing (a panel of 55 genes, listed in Table S4) on 305 patients. WES (n=25, divided into five groups) and WGS (n=17) were performed on 42 randomly selected tumor samples and their matched peripheral blood, to build a somatic mutation calling principle and to filter out putative germline variants. Sanger sequencing was used to confirm somatic mutations not observed in matched peripheral blood mononuclear cells (PBMCs).

For WGS, DNA was sheared to about 300 bp fragments by Covaris DNA shearing system. After end-repaired and 3’-ends adenylated, Illumina PE adapters were ligated to DNA fragments to generate indexed library. Library was validated by Agilent 2100 Bioanalyzer and sequencing was performed on Illumina HiSeq platform with 150 bp paired-end strategy in WuXi NextCODE, Shanghai. For WES, exome regions were captured by a SeqCap EZ Human Exome kit (version 3.0) and sequencing was performed on HiSeq 4000 platform with 150 bp paired-end strategy in Righton, Shanghai. WES (n=25, divided into five groups) and WGS (n=17) were performed on 42 randomly selected tumor samples and their matched peripheral blood, to build a somatic mutation calling principle and to filter out putative germline variants. Sanger sequencing was used to confirm somatic mutations not observed in matched PBMCs. For targeted sequencing, polymerase chain reaction (PCR) primers were designed by Primer 5.0 software. Multiplexed libraries of tagged amplicons from tumor samples were generated in Righton, Shanghai. Deep sequencing was performed using established Illumina protocols on HiSeq 4000 platform (Illumina). Genome Analysis Toolkit (GATK) Haplotype Caller and GATK Unified Genotyper were applied to call single-nucleotide variants (SNVs) and indels. SNVs and indels were mapped to the genome location using the UCSC Genome Browser (http://genome.ucsc.edu). All the somatic functional mutations, including nonsynonymous SNVs, frameshift or in-frame indels, stopgain or stoploss were obtained. Visual inspection was used to exclude potential false positive results. Homemade pipeline was used to filter SNVs and indels detected by the above software, excluding: 1) mutations reported with low confidence; 2) germline mutations detected from control samples; 3) population-related variants reported in 1000 Genomes (dbSNP 137) as common SNPs and not included in COSMIC (the Catalogue of Somatic Mutations in Cancer) version v77.

Genetic subtypes of patients were stratified by a simplified classification algorithm,^4^ which assigned patients into *TP53*^mut^, MCD-like, N1-like, BN2-like, ST2-like, EZB-like, or “not otherwise specified” (NOS) subtypes.

**RNA sequencing (RNA-seq) and functional analysis**

Total RNA was extracted using Trizol and RNeasy Mini Kit (Qiagen, Hilden, Germany) from frozen samples and qualified for RNA-seq in all 590 patients, among which 186 were referred in our previous study.^5^ RNA purification, reverse transcription, library construction and sequencing were performed in WuXi NextCODE according to the manufacturer's instructions (Illumina). PolyA mRNA was purified from total RNA using oligo-dT-attached magnetic beads and then fragmented by fragmentation buffer. The synthesized cDNA was subjected to end-repair, phosphorylation and ‘A’ base addition according to Illumina’s library construction protocol. Then Illumina sequencing adapters were added to both size of the cDNA fragments. After PCR amplification for DNA enrichment, the target fragments of 200-300 bp were cleaned up. After library construction, Qubit (Thermo Fisher Scientific) was used to quantify concentration of the resulting sequencing libraries, while the size distribution was analyzed using Agilent BioAnalyzer 2100 (Agilent). After library validation, Illumina cBOT cluster generation system with HiSeq PE Cluster Kits (Illumina) was used to generate clusters. Paired-end sequencing was performed using an Illumina HiSeq system following Illumina-provided protocols for 2×150 paired-end sequencing. Read pairs were aligned to Human Reference Genome version hg19 (downloaded from UCSC Genome Browser, http://genome.ucsc.edu/) by Burrows-Wheeler Aligner (BWA) version 0.7.13-r1126. Samtools version 1.3 was used to generate chromosomal coordinate-sorted bam files and to remove PCR duplications. The reads were then realigned around potential indel regions by GATK version 3.4 IndelRealigner with the recommended pipeline. Apart from the Hans algorithm,^1^ COO classification was determined by Lymph2Cx assay.^6^

Raw read counts were generated by a Python framework named HTSeq.^7^ R package “limma” (version 3.44.3)^8^ was used to remove batch effects, normalize raw data, and obtain differentially expressed genes. GSEA was performed by R package “clusterProfiler” (version 3.16.1),^9^ using annotated gene sets collected from Molecular Signatures Database (MSigDB),^10,11^ and a published online database (https://lymphochip.nih.gov/signaturedb/). The MSigDB gene sets (http://www.gsea-msigdb.org/gsea/msigdb/index.jsp) are divided into 9 major collections, i.e., C1-C9. Among them, C2 indicates curated gene sets from online pathway databases, publications in PubMed, and knowledge of domain experts, and C5 indicates ontology gene sets consisting of genes annotated by the same ontology term. Kyoto Encyclopedia of Genes and Genomes (KEGG) pathways from C2 (c2.cp.kegg.v7.4.symbols.gmt) and Gene Ontology (GO) Biological Process terms from C5 (c5.go.bp.v7.4.symbols.gmt) were applied.

The levels of infiltrating immune and stromal cells were calculated by online tools TIMER2.0 based on EPIC, MCPCOUNTER, TIMER, and xCell computational methods (http://timer.cistrome.org),^12^ performing cell type enrichment analysis from gene expression data for immune and stroma cell types and presenting comprehensive collection of gene expression enrichment scores for different cell types.

**Protein-protein interaction network analysis**

The STRING database^13^ was applied to construct the interaction network of core genes from specified pathways in each DEL subtype. Data were subsequently put into Cytoscape (version 3.9.1)^14^ for network integration and visualization. To deeply excavate the relationship between the key pathways in each subtype, the top 30 genes in each pathway network (ranked by Maximal Clique Centrality algorithms^14^) were used as core genes for displaying protein-protein interactions. Each core gene was marked as one node, and combined scores (range from 0 to 1) of each pair of node connection were obtained from The STRING database. The cutoff value of combined scores was 0.4. Combined scores range from 0.4 to 0.7, 0.7 to 0.9, and 0.9 to 1.0, suggested weak, moderate, and strong interaction, respectively.

**Unsupervised consensus clustering for molecular subtyping**

Unsupervised clustering of gene expression data was applied for novel subtype discovery of DEL. To improve the robustness of clustering, we used a consensus partitioning approach implemented in the R package “cola”.^15^ Non-protein-coding genes and genes on sex chromosomes were removed before using R package “cola”.^15^ Data preprocessing removed genes with very small variance from the matrix. Four top-value methods in “cola” are listed as follows: standard deviation (SD), median absolute deviation (MAD), coefficient of variation (CV) and ability to correlate to other rows (ATC). ATC, a new method provided by “cola”, was applied for feature selection. Top *n* (*n*=1000, 2000, or 3000) genes with the highest ATC scores were then selected for partitioning. Six partitioning methods in “cola” are listed as follows: hierarchical clustering (hclust), k-means clustering (kmeans), spherical k-means clustering (skmeans), partitioning around medoids (pam), model-based clustering (mclust) and non-negative matrix factorization (NMF). Spherical k-means (skmeans) clustering was adopted for its reliability and stability compared to other designated partitioning methods. The optional best number of subgroups was determined by metrics including 1-PAC (proportion of ambiguous clustering) score, silhouette score, etc.

The consensus sub-clusters were obtained from *a* (resampling iterations of the clustering, usually *a*=50 to 1000), by randomly selecting a fraction of the samples (usually 80%). The best or optional best number of subgroups *k* (usually *k*=2 to 6) was determined by metrics including 1-PAC (the proportion of ambiguous clustering) score, silhouette score, concordance, and Jaccard index. Above cited and reorganized from Gu et al.^15^

**Cell transfection**

B‐cell lymphoma cell line SU-DHL-4 was obtained from American Type Culture Collection (Manassas, VA, USA). Viral particles containing puriﬁed plasmids expressing pGV358/GFP/Puro-*KMT2D* (NM-003482, residues 4839-5537, containing SET domain, wild-type, wt), and pGV358/GFP/Puro-*KMT2D* (NM-003482, residues 4839-5537, containing SET domain, R5432Q) were used to generate SU-DHL-4 *KMT2D*^wt^ and SU-DHL-4 *KMT2D*^R5432Q^ cells. The supernatant fraction of HEK-293T cell cultures was condensed to a viral concentration of approximately 2×10^8^ transducing units/ml. The lentiviral particles were incubated with SU-DHL-4 cells for 72 hours with addition of polybrene (8 μg/ml). The stably transduced clones were selected by green and/or red fluorescence protein using flow cytometry or puromycin treatment for two weeks. DLBCL cell line SU-DHL-4 showing high BCL2 and MYC expression transfected with plasmid containing KMT2D (*KMT2D*^R5432Q^) represented typical C1 subtype of DEL.

***In vitro* co‐culture system**

PBMCs were isolated from a healthy donor using ficoll density gradient centrifugation, with a mixed population of myeloid and lymphoid cells including B cells (~15%), T cells (~70%), monocytes (~5%), and natural killer cells (~10%). Effector (E) to target (T) ratio is defined as ratio of number of PBMCs to lymphoma cells. The E:T ratio was 5:1, as previously suggested.^16^ PBMCs were grown in RPMI-1640 medium with 10% heat-inactivated fetal bovine serum and 1% penicillin/streptomycin (15140122, Gibco, Carlsbad, CA, USA) in a humidified atmosphere containing 95% air-5% CO_2_ at 37 °C. Cell lines were treated with tucidinostat (5 μM) and doxorubicin (200 nM) for 48 hours based on clinically achievable concentrations used in CR-CHOP (tucidinostat plus rituximab, cyclophosphamide, doxorubicin, vincristine, prednisone) regimen according to previous studies.^17,18^

**Flow cytometry**

To detect percentage of immune cells, co‐cultured cells were stained with anti-CD3 (BV786, BD Pharmingen), anti-CD4 (FITC, BD Pharmingen), anti-CD8 (PerCP-Cy5.5, BD Pharmingen), anti-CD14 (FITC, BD Pharmingen), anti-CD68 (PE-Cy7, BD Pharmingen), anti-CD206 (APC, BD Pharmingen), anti-LAG-3 (APC-R700, BD Pharmingen), anti-PD-1 (BV421, BioLegend), and anti-TIM-3 (BV605, BioLegend). Data were analyzed using Flowjo software (Becton Dickinson).

**Establishment and maintenance of patient-derived xenograft models**

Four-week-old female NOD/LtSz-scid IL2 receptor γ-/- (NSG) and NOD-SCID mice were obtained from Shanghai Laboratory Animal Center (Shanghai, China) for the establishment and maintenance of patient-derived xenograft (PDX) models. Tumor samples of DLBCL patients were collected by ultrasound-guided core needle biopsy and stored in 4°C Hanks balanced salt solution supplemented with antibiotics. Each fresh tumor sample was cut into ~3 mm^3^ pieces and kept in sterile Hanks balanced salt solution until xenotransplantation. Three to six female NSG mice were implanted with each primary tumor sample. Ketamine/xylazine cocktail at a ratio of 100 mg/kg:20 mg/kg was used to anesthetize mice. After xenotransplantation, animals were evaluated regularly and euthanized when tumors exceeded 12 mm in any dimension. Heterotopic PDX models were maintained by passaging tumor tissue directly from NSG to NOD-SCID mice.

**Assay for Transposase Accessible Chromatin sequencing**

For each Assay for Transposase Accessible Chromatin sequencing (ATAC-seq) experiment, 50000 nuclei were used as input according to Corces et al.^19^ Each ATAC-seq library was sequenced by Illumina HiSeq 2000 at the depth of 40-60 million per sample (150 bp paired-end reads). Raw sequencing reads were aligned against the human reference genome hg19 using Bowtie 2 (version 2.2.9)^20^ with default settings. PCR duplicates were removed by SAMtools (version 1.3.1).^21^ ATAC-seq fragment length distribution in peaks was examined using ataqv (version 1.2.1).^22^ RPKM (Reads Per Kilobase per Million mapped reads)-normalized bigwig files were generated by deepTools (version 3.3.1)^23^ for visualization with Integrative Genomics Viewer (IGV).^24^ Peaks were called by MACS2 (2.1.0.20151222)^25^ using the following parameters: -f BAM -g hs -q 0.01 --keep-dup all. Peak summits were extended 250 bp in both directions, followed by removal of ENCODE blacklisted regions using BEDTools (version 2.29.0).^26^ Distal ATAC-seq peaks were those that did not overlap with regions at transcription start sites +/-100bp (hg19 reference genes). The distal peaks from all samples were merged to generate a master bed file to calculate the reads that overlap with each peak. The resulted count table (peaks by samples) was used as input for the R package DESeq2 (version 1.32.0)^27^ for normalization and differential accessibility analysis between samples. The peaks with less than 200 reads in all samples were deemed as weak peaks and discarded. Differential peaks were those with adjusted p<0.05. GO and pathway associated with differentially accessible regions were annotated by Genomic Regions Enrichment of Annotations Tool (GREAT).^28^ The peak count table was also used as input for ChromVAR (version 1.40.0) analysis using JASPAR TF motif collection.^29^

**Quantitative real-time PCR (RT-PCR)**

The total RNA was extracted using Trizol reagent, and complementary DNA was synthesized using Prime Script RT Reagent Kit with gDNA Eraser (TaKaRa, Dalian, China). Quantitative RT-PCR was performed using SYBR Premix Ex TaqTM II (TaKaRa) and ABI ViiA7 (Applied Biosystems, Foster City, CA, USA). Relative quantification was calculated using the 2−ΔΔCT method. The primer sequences of selected genes are listed in the Table S5, and *GAPDH* was used as endogenous control. SU-DHL-4 cells were used for calibration.

**Statistical analysis**

Two-tailed t-test or one-way analysis of variance were used for comparing the means of continuous variables between groups when the data followed normal distribution and equal variances. Mann-Whitney U or Kruskal-Wallis H test were used as the nonparametric equivalent of the aforementioned methods. Chi-square or Fisher’s exact tests were used to evaluate the association of non-ordinal categoric variables. As for survival analysis, progression-free survival (PFS) was measured from diagnosis to the date of documentation of either disease progression or death from any cause, and overall survival (OS) was measured from diagnosis to the date of death from any cause. Survival distribution was estimated using the Kaplan-Meier method and compared by the log-rank test. A two-sided p<0.05 was considered statistically significant. Statistical analyses and data visualization were performed using Statistical Package for the Social Sciences software (version 25.0), GraphPad Prism (version 8.0.1) and R studio (version 4.0.1).

**References**

1. Hans CP, Weisenburger DD, Greiner TC, Gascoyne RD, Delabie J, Ott G, et al. Confirmation of the molecular classification of diffuse large B-cell lymphoma by immunohistochemistry using a tissue microarray. *Blood* 2004; **103**(1): 275-82.

2. Swerdlow SH, Campo E, Pileri SA, Harris NL, Stein H, Siebert R, et al. The 2016 revision of the World Health Organization classification of lymphoid neoplasms. *Blood* 2016; **127**(20): 2375-90.

3. Meriranta L, Pasanen A, Alkodsi A, Haukka J, Karjalainen-Lindsberg ML, Leppa S. Molecular background delineates outcome of double protein expressor diffuse large B-cell lymphoma. *Blood Adv* 2020; **4**(15): 3742-53.

4. Shen R, Fu D, Dong L, Zhang MC, Shi Q, Shi ZY, et al. Simplified algorithm for genetic subtyping in diffuse large B-cell lymphoma. *Signal Transduct Target Ther* 2023; **8**(1): 145.

5. Fang Y, Zhang MC, Xu PP, Zhang SJ, Wang L, Cheng S, et al. Integrative genome-wide chromatin accessibility and transcriptome profiling of diffuse large B-cell lymphoma. *Clin Transl Med* 2022; **12**(7): e975.

6. Scott DW, Wright GW, Williams PM, Lih CJ, Walsh W, Jaffe ES, et al. Determining cell-of-origin subtypes of diffuse large B-cell lymphoma using gene expression in formalin-fixed paraffin-embedded tissue. *Blood* 2014; **123**(8): 1214-7.

7. Anders S, Pyl PT, Huber W. HTSeq--a Python framework to work with high-throughput sequencing data. *Bioinformatics* 2015; **31**(2): 166-9.

8. Ritchie ME, Phipson B, Wu D, Hu Y, Law CW, Shi W, et al. limma powers differential expression analyses for RNA-sequencing and microarray studies. *Nucleic Acids Res* 2015; **43**(7): e47.

9. Yu G, Wang LG, Han Y, He QY. clusterProfiler: an R package for comparing biological themes among gene clusters. *OMICS* 2012; **16**(5): 284-7.

10. Subramanian A, Tamayo P, Mootha VK, Mukherjee S, Ebert BL, Gillette MA, et al. Gene set enrichment analysis: a knowledge-based approach for interpreting genome-wide expression profiles. *Proc Natl Acad Sci U S A* 2005; **102**(43): 15545-50.

11. Liberzon A, Birger C, Thorvaldsdottir H, Ghandi M, Mesirov JP, Tamayo P. The Molecular Signatures Database (MSigDB) hallmark gene set collection. *Cell Syst* 2015; **1**(6): 417-25.

12. Li T, Fu J, Zeng Z, Cohen D, Li J, Chen Q, et al. TIMER2.0 for analysis of tumor-infiltrating immune cells. *Nucleic Acids Res* 2020; **48**(W1): W509-W14.

13. Szklarczyk D, Gable AL, Nastou KC, Lyon D, Kirsch R, Pyysalo S, et al. The STRING database in 2021: customizable protein-protein networks, and functional characterization of user-uploaded gene/measurement sets. *Nucleic Acids Res* 2021; **49**(D1): D605-D12.

14. Shannon P, Markiel A, Ozier O, Baliga NS, Wang JT, Ramage D, et al. Cytoscape: a software environment for integrated models of biomolecular interaction networks. *Genome Res* 2003; **13**(11): 2498-504.

15. Gu Z, Schlesner M, Hubschmann D. cola: an R/Bioconductor package for consensus partitioning through a general framework. *Nucleic Acids Res* 2021; **49**(3): e15.

16. Dijkstra KK, Cattaneo CM, Weeber F, Chalabi M, van de Haar J, Fanchi LF, et al. Generation of Tumor-Reactive T Cells by Co-culture of Peripheral Blood Lymphocytes and Tumor Organoids. *Cell* 2018; **174**(6): 1586-98 e12.

17. Ji MM, Huang YH, Huang JY, Wang ZF, Fu D, Liu H, et al. Histone modifier gene mutations in peripheral T-cell lymphoma not otherwise specified. *Haematologica* 2018; **103**(4): 679-87.

18. Clozel T, Yang S, Elstrom RL, Tam W, Martin P, Kormaksson M, et al. Mechanism-based epigenetic chemosensitization therapy of diffuse large B-cell lymphoma. *Cancer Discov* 2013; **3**(9): 1002-19.

19. Corces MR, Buenrostro JD, Wu B, Greenside PG, Chan SM, Koenig JL, et al. Lineage-specific and single-cell chromatin accessibility charts human hematopoiesis and leukemia evolution. *Nat Genet* 2016; **48**(10): 1193-203.

20. Langmead B, Salzberg SL. Fast gapped-read alignment with Bowtie 2. *Nat Methods* 2012; **9**(4): 357-9.

21. Li H, Handsaker B, Wysoker A, Fennell T, Ruan J, Homer N, et al. The Sequence Alignment/Map format and SAMtools. *Bioinformatics* 2009; **25**(16): 2078-9.

22. Orchard P, Kyono Y, Hensley J, Kitzman JO, Parker SCJ. Quantification, Dynamic Visualization, and Validation of Bias in ATAC-Seq Data with ataqv. *Cell Syst* 2020; **10**(3): 298-306 e4.

23. Ramirez F, Dundar F, Diehl S, Gruning BA, Manke T. deepTools: a flexible platform for exploring deep-sequencing data. *Nucleic Acids Res* 2014; **42**(Web Server issue): W187-91.

24. Robinson JT, Thorvaldsdottir H, Winckler W, Guttman M, Lander ES, Getz G, et al. Integrative genomics viewer. *Nat Biotechnol* 2011; **29**(1): 24-6.

25. Liu T. Use model-based Analysis of ChIP-Seq (MACS) to analyze short reads generated by sequencing protein-DNA interactions in embryonic stem cells. *Methods Mol Biol* 2014; **1150**: 81-95.

26. Quinlan AR, Hall IM. BEDTools: a flexible suite of utilities for comparing genomic features. *Bioinformatics* 2010; **26**(6): 841-2.

27. Love MI, Huber W, Anders S. Moderated estimation of fold change and dispersion for RNA-seq data with DESeq2. *Genome Biol* 2014; **15**(12): 550.

28. McLean CY, Bristor D, Hiller M, Clarke SL, Schaar BT, Lowe CB, et al. GREAT improves functional interpretation of cis-regulatory regions. *Nat Biotechnol* 2010; **28**(5): 495-501.

29. Mathelier A, Fornes O, Arenillas DJ, Chen CY, Denay G, Lee J, et al. JASPAR 2016: a major expansion and update of the open-access database of transcription factor binding profiles. *Nucleic Acids Res* 2016; **44**(D1): D110-5.

**Supplementary Figures**

**Shi, et al. Supplementary Figure 1**


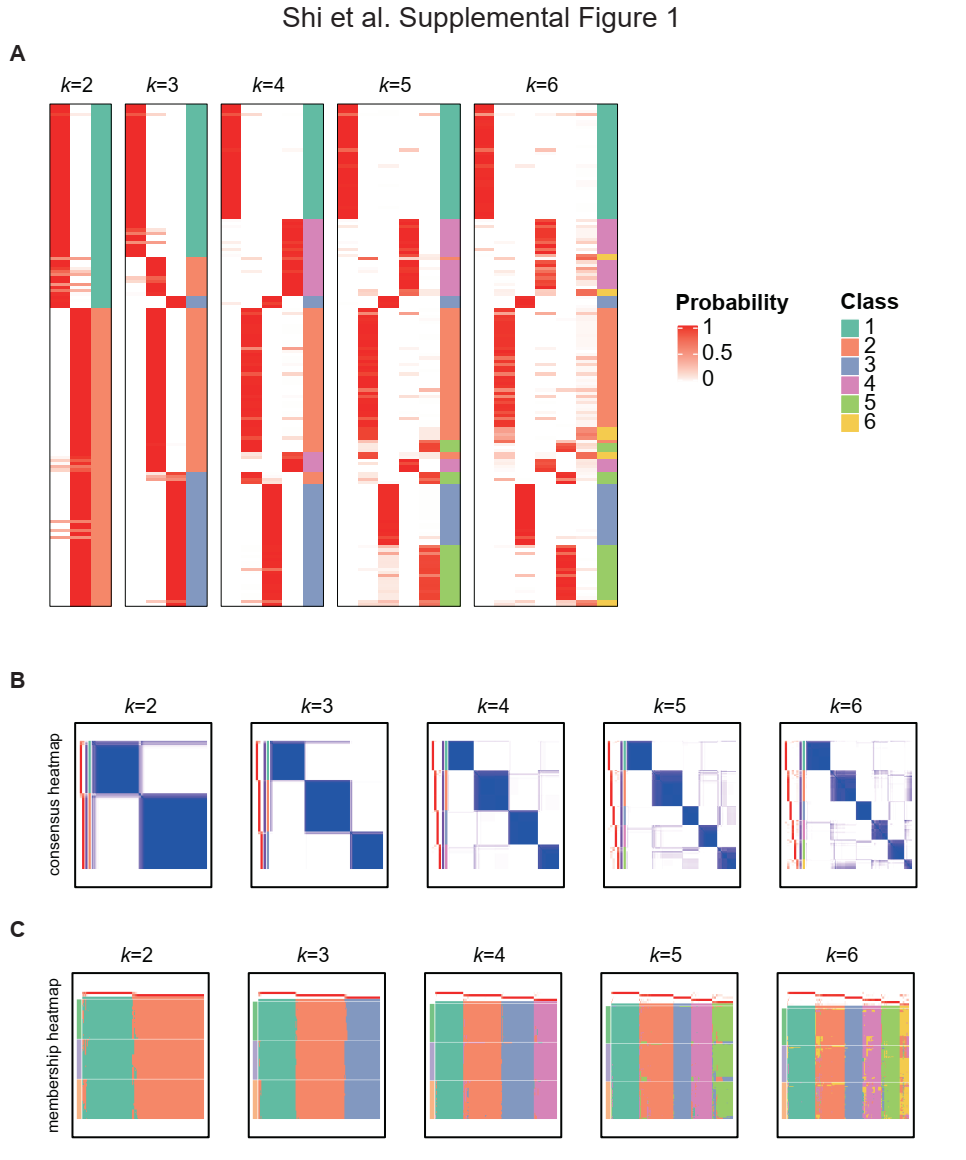


**Supplementary Figure 1. Decision of the best or optimal best cluster number *k*.**

(A) Consensus classes showed how subgroups were corresponded with increasing *k* (*k*=2, 3, 4, 5, or 6). (B) Consensus heatmaps at each *k* (*k*=2, 3, 4, 5, or 6). (C) Membership matrices at each *k* (*k*=2, 3, 4, 5, or 6).

**Shi, et al. Supplementary Figure 2**


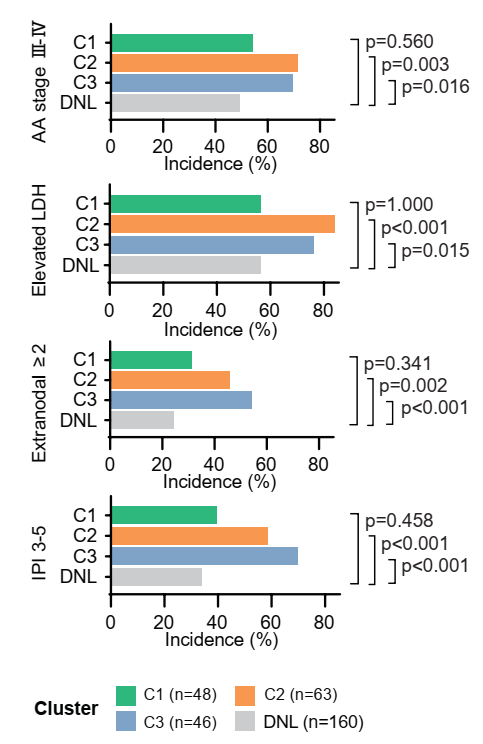


**Supplementary Figure 2. Comparison of clinical indicators between each DEL subtype and DNL.** P values calculated with Chi-square tests were shown.

DEL indicates double expressor lymphoma, and DNL indicates double negative lymphoma.

**Shi, et al. Supplementary Figure 3**


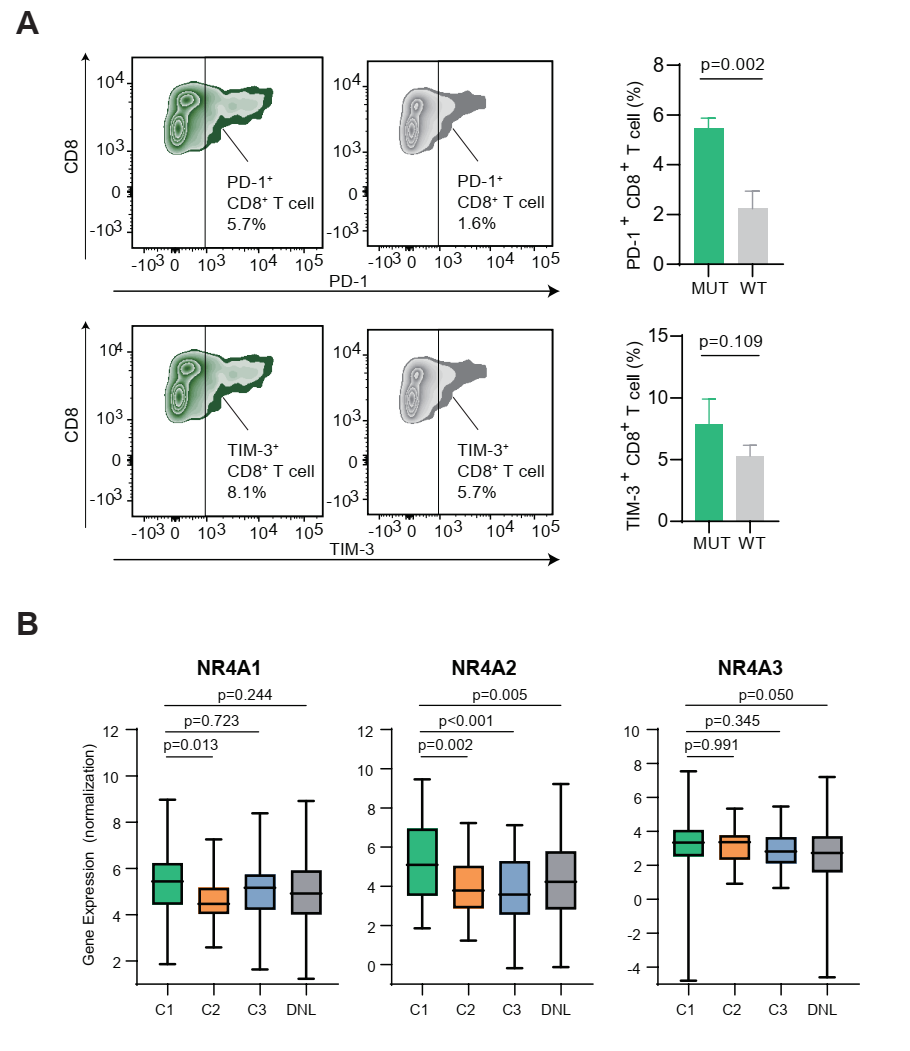


**Supplementary Figure 3. Transcriptional and microenvironmental alterations in C1 subtype of DEL.**

(A) Flow cytometry analysis of proportion of PD-1^+^ CD8^+^ T cells and TIM-3^+^ CD8^+^ T cells. Co-culture of PBMC and SU-DHL-4 *KMT2D*^mut^ cells, and co-culture of PBMC and SU-DHL-4 *KMT2D*^wt^ cells. (B) Comparison of gene expression level of TFs of NR4A family in patients of C1, C2, C3, and DNL.

DNL indicates double negative lymphoma; GSEA, gene set enrichment analysis; MUT, mutant; NES, normalized enrichment score; NF-κB, NF-kappa B; PBMC, peripheral blood mononuclear cell; TCR, T cell receptor; and WT, wild-type.

**Shi, et al. Supplementary Figure 4**


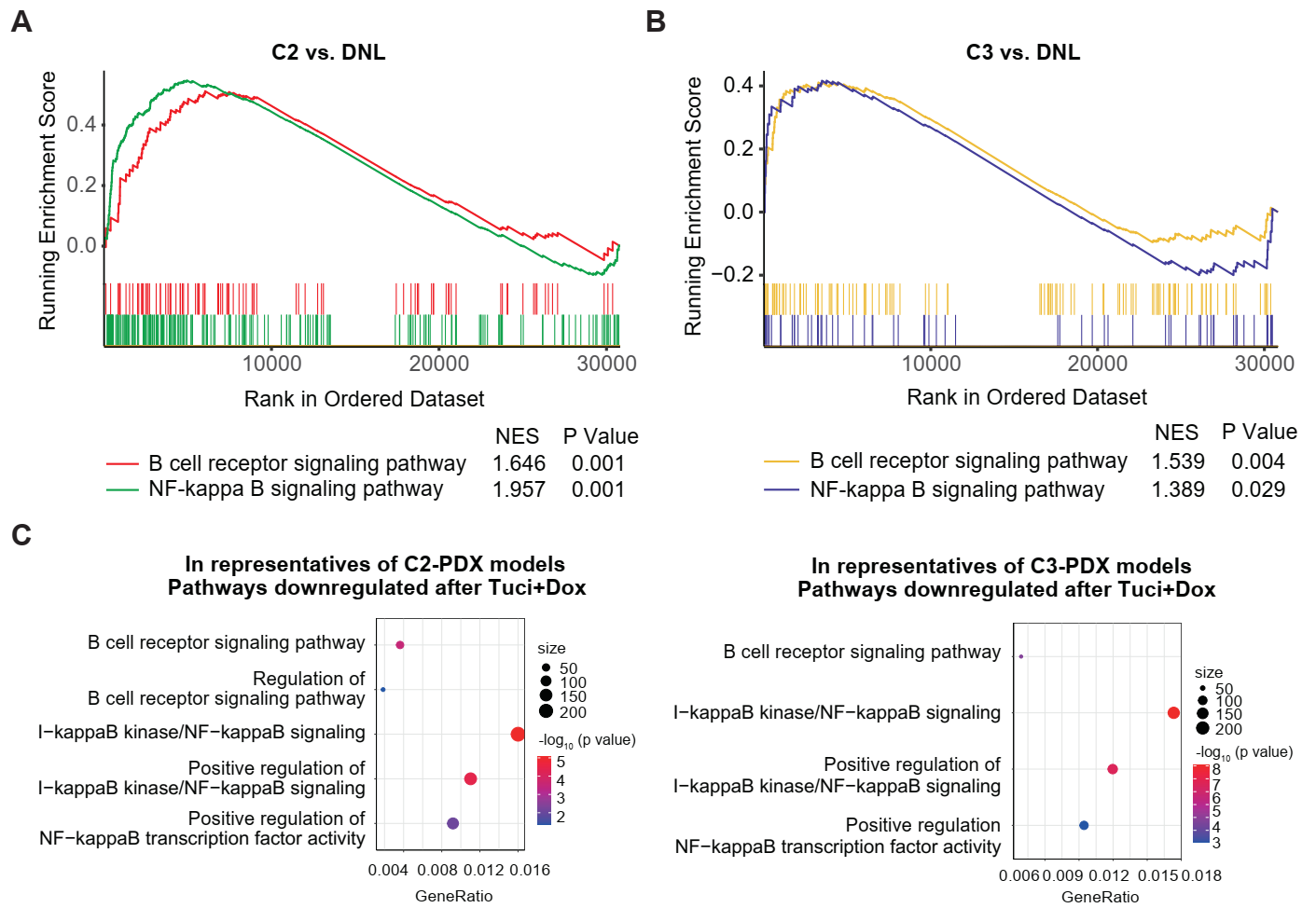


**Supplementary Figure 4. RNA-seq and ATAC-seq analysis of C2-, C3-, and DNL-PDX models.**

(A) GSEA analysis exhibited upregulation of BCR and NF-κB signaling pathway in C2-PDX models, compared with DNL-PDX models. (B) GSEA analysis exhibited upregulation of BCR and NF-κB signaling pathway in C3-PDX models, compared with DNL-PDX models. (C) Downregulated pathways in C2-PDX models (left panel) and C3-PDX models (right panel) detected by ATAC-seq after tucidinostat and doxorubicin treatment.

ATAC-seq indicates assay for transposase-accessible chromatin using sequencing; BCR, B-cell receptor; DEL, double expressor lymphoma; DNL, double negative lymphoma; GSEA, gene set enrichment analysis; NF-κB, NF kappa-B; PDX, patient-derived xenograft; and RNA-seq, RNA sequencing.

**Supplementary Tables**

**Supplementary Table 1. Decision of the best** **or optimal best cluster number *k* based on the following statistics.**

| ***k*** | **Jaccard index** | **1-PAC score** | **Mean silhouette** | **Concordance** |
| --- | --- | --- | --- | --- |
| 2 | 0.514 | 0.960 | 0.934 | 0.975 |
| 3 | 0.472 | 0.990 | 0.959 | 0.984 |
| 4 | 0.600 | 0.977 | 0.959 | 0.981 |
| 5 | 0.668 | 0.930 | 0.915 | 0.950 |
| 6 | 0.815 | 0.890 | 0.836 | 0.912 |

PAC indicates proportion of ambiguous clustering.

Footnotes: Following statements are the suggested rules provided by the R package “cola” (https://bioconductor.org/packages/cola/) for the best or optimal best *k*:

All *k* with Jaccard index larger than 0.95 are removed because increasing *k* does not provide enough extra information.

For all *k* with 1-PAC score larger than 0.9, the maximal *k* is taken as the best *k*, and other *k* are marked as optional *k*.

If it does not fit the second rule. The *k* with the maximal vote of the highest 1-PAC score, highest mean silhouette, and highest concordance is taken as the best *k*.

**Supplementary Table 2.** Clinical and pathological characteristics of DEL and DNL.

| Characteristics | DEL  (n=157) | DNL  (n=160) | P value  (DEL vs. DNL) |
| --- | --- | --- | --- |
|  | **No. (%)** | **No. (%)** |  |
| Male | 86 (54.8) | 102 (63.7) | 0.104 |
| Age >60 | 95 (60.5) | 73 (45.6) | 0.008 |
| Ann Arbor stage Ⅲ-Ⅳ | 103 (65.6) | 79 (49.4) | 0.003 |
| ECOG ≥2 | 29 (18.5) | 13 (8.1) | 0.007 |
| Elevated serum LDH | 115 (73.2) | 90 (56.3) | 0.002 |
| Extranodal sites ≥2 | 69 (43.9) | 39 (24.4) | <0.001 |
| IPI 3-5 | 88 (56.1) | 54 (33.8) | <0.001 |
| Cell of origin (determined by the Hans algorithm) | | | |
| GCB | 35/152 (23.0) | 74/154 (48.1) | <0.001 |
| non-GCB | 117/152 (77.0) | 80/154 (51.9) |  |
| Cell of origin (determined by the Lymph2Cx assay) | | | |
| ABC | 112 (71.3) | 49 (30.6) | <0.001 |
| GCB | 24 (15.3) | 67 (41.9) |  |
| Unclassified | 21 (13.4) | 44 (27.5) |  |
| IHC CD5 positive | 25/136 (18.4) | 6/142 (4.2) | <0.001 |
| FISH *BCL2* positive | 8 (5.1) | 6 (3.8) | 0.560 |
| FISH *BCL6* positive | 41 (26.1) | 25 (15.6) | 0.021 |
| FISH *MYC* positive | 7 (4.5) | 5 (3.1) | 0.534 |

Values represent n (%) of patients unless otherwise indicated.

ABC indicates activated B-cell; DEL, double expressor lymphoma; DNL, double negative lymphoma; ECOG, Eastern Cooperative Oncology Group; FISH, Fluorescence in situ hybridization; GCB, germinal center B-cell; IHC, immunohistochemistry; IPI, international prognostic index; and LDH, lactate dehydrogenase.

**Supplementary Table 3.** Clinicopathological characteristics according to subtypes of DEL.

| Characteristics | C1  (n=48) | C2  (n=63) | C3  (n=46) | P value  (C1 vs. C2 vs. C3) |
| --- | --- | --- | --- | --- |
|  | **No. (%)** | **No. (%)** | **No. (%)** |  |
| Male | 22 (45.8) | 34 (54.0) | 30 (65.2) | 0.166 |
| Age >60 | 28 (58.3) | 37 (58.7) | 30 (65.2) | 0.739 |
| Ann Arbor stage Ⅲ-Ⅳ | 26 (54.2) | 45 (71.4) | 32 (69.6) | 0.132 |
| ECOG ≥2 | 6 (12.5) | 14 (22.2) | 9 (19.6) | 0.414 |
| Elevated serum LDH | 27 (56.3) | 53 (84.1) | 35 (76.1) | 0.004 |
| Extranodal sites ≥2 | 15 (31.3) | 29 (46.0) | 25 (54.3) | 0.072 |
| IPI 3-5 | 19 (39.6) | 37 (58.7) | 32 (69.6) | 0.012 |
| Cell of origin (determined by the Hans algorithm) | | | | |
| GCB | 12/46 (26.1) | 15/62 (24.2) | 8/44 (18.2) | 0.646 |
| non-GCB | 34/46 (73.9) | 47/62 (75.8) | 36/44 (81.8) |  |
| Cell of origin (determined by the Lymph2Cx assay) | | | | |
| ABC | 17 (35.4) | 56 (88.9) | 39 (84.8) | <0.001 |
| GCB | 15 (31.3) | 7 (11.1) | 2 (4.3) |  |
| Unclassified | 16 (33.3) | 0 (0.0) | 5 (10.9) |  |
| IHC CD5 positive | 6/39 (15.4) | 14/56 (25.0) | 5/41 (12.2) | 0.233 |
| FISH *BCL2* positive | 4 (8.3) | 2 (3.2) | 2 (4.3) | 0.541 |
| FISH *BCL6* positive | 15 (31.3) | 19 (30.2) | 7 (15.2) | 0.134 |
| FISH *MYC* positive | 1 (2.1) | 4 (6.3) | 2 (4.3) | 0.565 |

Values represent n (%) of patients unless otherwise indicated.

ECOG indicates Eastern Cooperative Oncology Group; FISH, Fluorescence in situ hybridization; GCB, germinal center B-cell-like; IPI, international prognostic index; and LDH, lactate dehydrogenase.

**Supplementary Table 4.** A panel of 55 genes.

| *ARID1A* | *CD79B* | *FOXO1* | *MTOR* | *STAT3* |
| --- | --- | --- | --- | --- |
| *ATM* | *CIITA* | *GNA13* | *MYC* | *STAT6* |
| *B2M* | *CREBBP* | *HIST1H1C* | *MYD88* | *TBL1XR1* |
| *BCL6* | *DDX3X* | *HIST1H1E* | *NFKBIE* | *TET2* |
| *BTG1* | *DTX1* | *IRF4* | *NOTCH1* | *TMSB4X* |
| *BTG2* | *DUSP2* | *IRF8* | *NOTCH2* | *TNFAIP3* |
| *CARD11* | *EBF1* | *KMT2C* | *PIM1* | *TNFRSF14* |
| *CCND3* | *EP300* | *KMT2D* | *PRDM1* | *TP53* |
| *CD58* | *EZH2* | *LYN* | *PTPN6* | *TSC2* |
| *CD70* | *FAS* | *MAPK7* | *SGK1* | *ZFP36L1* |
| *CD79A* | *FBXW7* | *MPEG1* | *SOCS1* | *ZNF608* |

**Supplementary Table 5. Primer sequences used in quantitative real-time PCR**

| **Gene** | **Forward** | **Reverse** |
| --- | --- | --- |
| *BTK* | TCTGAAGCGATCCCAACAGAA | TGCACGGTCAAGAGAAACAGG |
| *NFKB1* | GAAGCACGAATGACAGAGGC | GCTTGGCGGATTAGCTCTTTT |
| *BLNK* | ACTCGGACTCAGAGATGTACG | GGCTTACTGGGAAGTGTCTTG |
| *MALT1* | CGCCTCAGTTGCCTAGACC | TCACCCATTAACTTCAGCAGAC |
| *PLCG2* | CATCCTATATGGCACTCAGTTCG | TCCTGGTGTAAGATTTTCAAGCC |
| *IKBKG* | CGGCAGAGCAACCAGATTCT | CCTGGCATTCCTTAGTGGCAG |
| *CARD11* | AACCTTCCAGGAGCGGTACTA | GTAGCGCATGGCTAAGTTGTA |
| *PRKCB* | AAACCTTGTACCTATGGACCCC | CCCAATCCCAAATCTCTACTGAC |
| *MAPK12* | CATGAGAAGCTAGGCGAGGAC | CAGCGTGGATATACCTCAGCC |
| *TRAF2* | CCTTCCCAGATAATGCTGCCC | GCTCTCGTATTCTTTCAGGGTC |
| *MAPK15* | GGGCCTATGGCATTGTGTG | TCTCTGGGCATCTGTCTTATCC |
| *DAXX* | GAGGCACGGTTGAAGCGTAA | CCATAGTCAGGGAAGGTATCAGG |
| *CCND1* | GCTGCGAAGTGGAAACCATC | CCTCCTTCTGCACACATTTGAA |
| *CCND2* | ACCTTCCGCAGTGCTCCTA | CCCAGCCAAGAAACGGTCC |
| *CCND3* | TACCCGCCATCCATGATCG | AGGCAGTCCACTTCAGTGC |
| *CDC25B* | GCATGGAGAGTCTCATTAGTGC | CTCCGCCTCCGCTTATTCT |
| *GAPDH* | GGAGCGAGATCCCTCCAAAAT | GGCTGTTGTCATACTTCTCATGG |
